# Effects of Variation in Al Content on the Emission of Eu Doped CaAlSiN_3_ Red Phosphor Synthesized by Combustion Synthesis Method for White LEDs

**DOI:** 10.3390/ijms222011301

**Published:** 2021-10-19

**Authors:** Shu-Chi Huang, Shyan-Lung Chung

**Affiliations:** 1 National Synchrotron Radiation Research Center, Hsinchu 30076, Taiwan; 2 Department of Chemical Engineering, National Cheng Kung University, Tainan 70101, Taiwan

**Keywords:** CaAlSiN_3_:Eu^2+^ phosphor, combustion synthesis method, LED

## Abstract

Effects of Al content on the formation and the photoluminescence properties of CaAlSiN_3_:Eu^2+^ phosphor (CASIN) were investigated by a combustion synthesis method. XRD (X-ray diffraction), combined with PL (photoluminescence), TEM-EDS (transmission electron microscope equipped with an energy-dispersive X-ray spectroscope), and SAED (selected area electron diffraction) measurements, show that the bar-like CASIN gives a stronger emission than the plate-like and agglomerated fine particles. The emission intensity increases as the Al content increased from Al = 0.2 to Al = 0.8, which resulted from the extent of formation of CASIN increases. Then, the emission intensity decreases as the Al content is increased from Al = 0.8 to Al = 1.5, which resulted from the transformation of morphology of CASIN and a large amount formation of AlN. In addition, the extent of formation of CASIN increases with increasing Al from Al = 0.2 to Al = 1.2 and begins to decrease as Al is further increased to 1.5, and thus the peak emission wavelength increases from 647 nm to 658 nm as the Al molar ratio is increased from 0.2 to 1.2 and begins to decrease when further increasing the Al molar ratio to 1.5, which resulted from the large amount of AlN formed.

## 1. Introduction

The white light-emitting diode (LED) has drawn much more attention because of high efficiency, long lifetime, compactness, environmental friendliness, and designable features. Generally, white LED lighting devices are fabricated based on the combination of an InGaN-based blue-LED chip and a yellow-emitting phosphor, i.e., YAG:Ce^3+^ [1,2]. One of the main problems of this type of LED lighting is the low color rendering index due to deficiency of red light. 

Besides the common requirements of high quantum efficiency, suitable emission colors and emission spectra, and high reliability, phosphors in the LED-based solid-state lighting are further required to have strong absorptions of ultraviolet or blue light and small thermal quenching. Therefore, searching for appropriate host lattice of LED phosphors has been a key topic. Fortunately, a new class of phosphors (i.e., rare-earth doped nitridosilicates) has been discovered and shown to be ideal for application in LED lighting due to their superior properties such as high quantum efficiency, red light emission, and high thermal and chemical stability [3]. Among them, CaAlSiN_3_:Eu^2+^ red-emitting phosphor materials have received great attention for excellent color rendition and thermal stability [4,5]. However, mostly articles reported the effects of Eu ion concentration in CaAlSiN_3_:Eu^2+^ red-emitting phosphor. Rarely, articles discuss the relation of Al metal concentration in CaAlSiN_3_:Eu^2+^ red-emitting phosphor, i.e., X-ray diffraction intensity, photoluminescence intensity, and crystal’s morphology of Al metal concentration in CaAlSiN_3_:Eu^2+^ red-emitting phosphor. 

S-X. Li et al. reported that the emission spectra had been blue shifted by increasing the Si/Al molar ratio and AlN-like secondary phase, which was always formed according to the stoichiometric composition with a Ca/Al/Si molar ratio of 1:1:1 to prepare CaAlSiN_3_-based red-emitting phosphors, even at high temperature and pressure, because of the low solubility of Al in CaAlSiN_3_ [4]. J. Yang et al. reported the Eu^2+^ doped CaAlSiN_3_ fabricated by an alloy-nitridation method, and the increase of Al/Si molar ratio makes red shift of emission band [5]. Y. W. Jung reported that the higher Al/Si ratio deactivated the high-energy component and a red-shift was observed [6]. 

In this article, we developed a CaAlSiN_3_:Eu^2+^ phosphor that can absorb the light from ultraviolet to visible light (i.e., 250–550 nm) and emit the orange-red light that the peak emission wavelength located at 647–658 nm. In addition, the advantages of this combustion synthesis method include the simple and low cost of equipment, short processing time, low N_2_ gas pressure, and reactants handled in ambient air. Thus, there is potential capability for mass production. After a series of experiments, we will discuss the relationship of X-ray diffraction intensity, photoluminescence intensity, morphology of particles, TEM analysis, and CIE chromaticity coordinates, which is compared with the commercial CaAlSiN_3_:Eu^2+^ phosphor.

## 2. Results and Discussion

### 2.1. Effects of Al Content on Product Formation and Morphology

**Figure 1:**
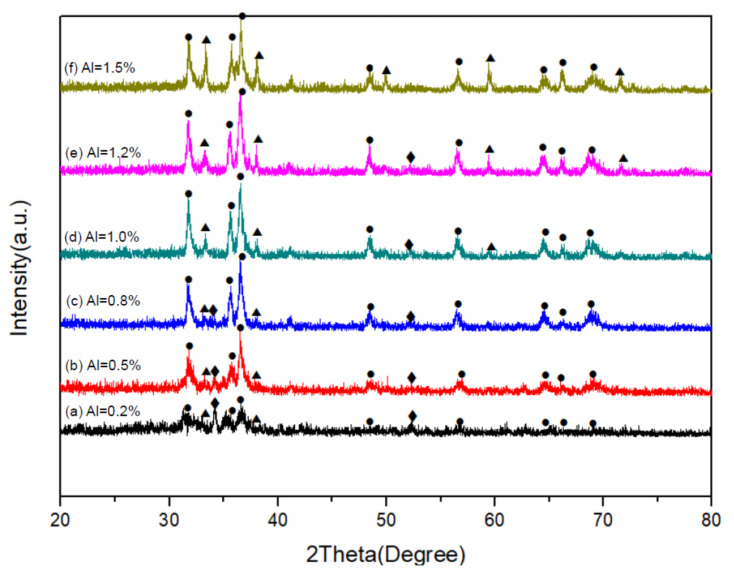
XRD patterns of the as-synthesized products obtained with Al = 0.2 (**a**); 0.5 (**b**); 0.8 (**c**); 1.0 (**d**); 1.2 (**e**); and 1.5 (**f**).

In Figure 1a–f are the XRD patterns of the as-synthesized products, obtained with various Al contents (Al = 0.2–1.5) in the reactant compacts. As can be seen, in addition to the phase of CASIN (JCPDS No.39-0747), AlN (JCPDS No. 89-3446) and residual Si (JCPDS No.79-0613) were also detected. As will be described later, the formation of AlN was confirmed by TEM-EDS and SAED measurements. The XRD peak intensity of CASIN increased with increasing Al to a maximum at Al = 1.2 but begins to decrease as Al is further increased to 1.5, indicating that the formation of CASIN continuously increases as the Al content is increased from 0.2 to 1.2 but begins to decrease as the Al content is further increased to 1.5. In addition, the formation of AlN continuously increases as the Al content is increased from 0.2 to 1.5. However, the residual Si decreases with increasing Al contents from Al = 0.2 to Al = 1.0, due to the formation of CASIN continuously increasing. Above Al = 1.0, the x-ray diffraction peak of residual Si cannot be detected. 

**Figure 2:**
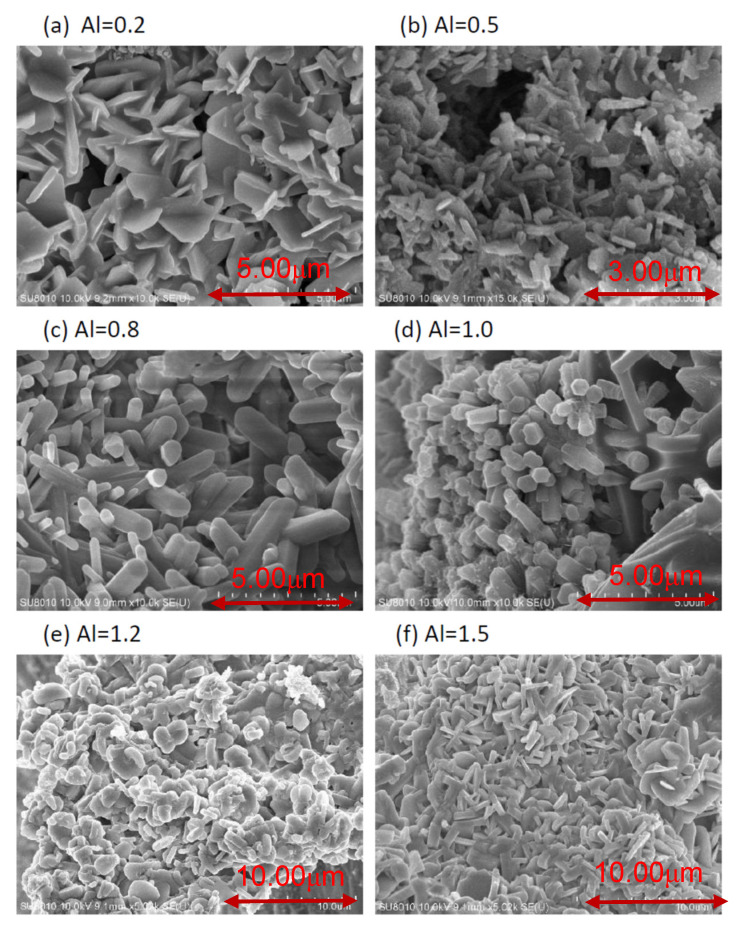
A typical SEM photograph of the products obtained with Al = 0.2 (**a**); 0.5 (**b**); 0.8 (**c**); 1.0 (**d**); 1.2 (**e**); and 1.5 (**f**).

After grinding with a mortar and pestle and washing with an acid, the products were observed to be composed mainly of bar-like, plate-like, and agglomerated fine particles. Figure 2 shows the SEM images of the as-synthesized products for Al = 0.2 to 1.5. As can be seen, when Al = 0.2, it is found by SEM observation that the plate-like particles are the major types of particles. With increasing Al to Al = 0.5, the number of plate-like particles decreases while that of bar-like particles increases, and at Al = 0.8 bar-like particles are the most abundant type of particles. As Al is increased from 0.8 to 1.5, the amount of plate-like and agglomerated fine particles increases, while that of bar-like particles greatly decreases.

**Figure 3:**
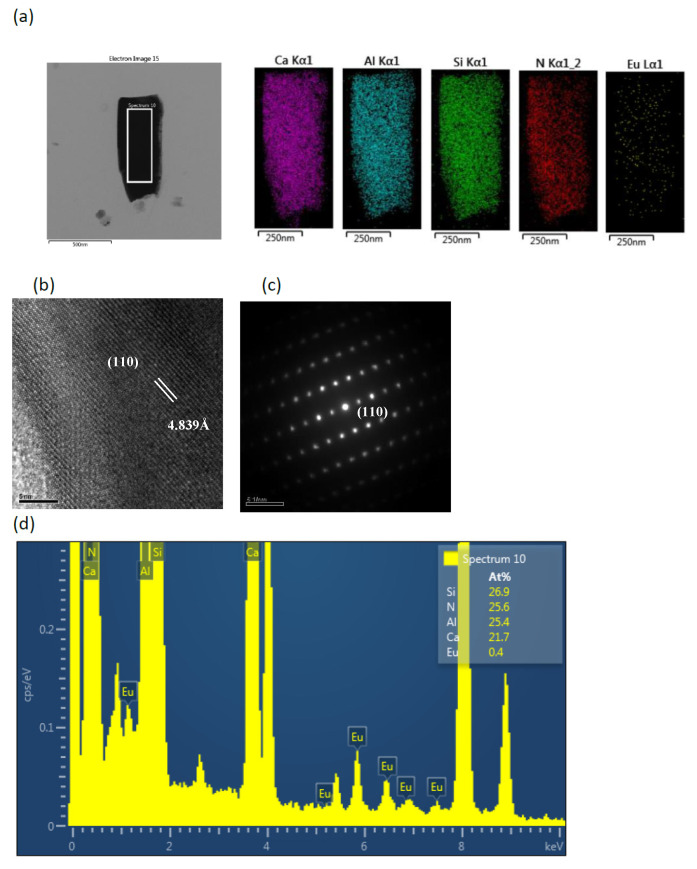
(**a**) TEM image and corresponding element mapping of Ca, Al, Si, N, Eu; (**b**) HRTEM image; (**c**) SAED pattern; and (**d**) element analysis of a rod-like particle (showing it to be CaAlSiN_3_:Eu^2+^) in a product synthesized with Ca:Al:Si:Si_3_N_4_:Eu_2_O_3_:NaN_3_:NH_4_Cl = 1.0:1.0:0.25:0.25:0.02:3.5:0.6.

**Figure 4:**
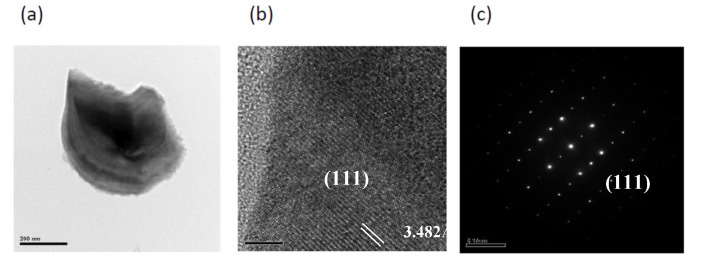
(**a**) TEM image; (**b**) HRTEM image; (**c**) SAED pattern of an agglomerated fine particles (showing it to be CaAlSiN_3_:Eu^2+^) in a product synthesized with Ca:Al:Si:Si_3_N_4_:Eu_2_O_3_:NaN_3_:NH_4_Cl = 1.0:0.8:0.25:0.25:0.02:3.5:0.6.

**Figure 5:**
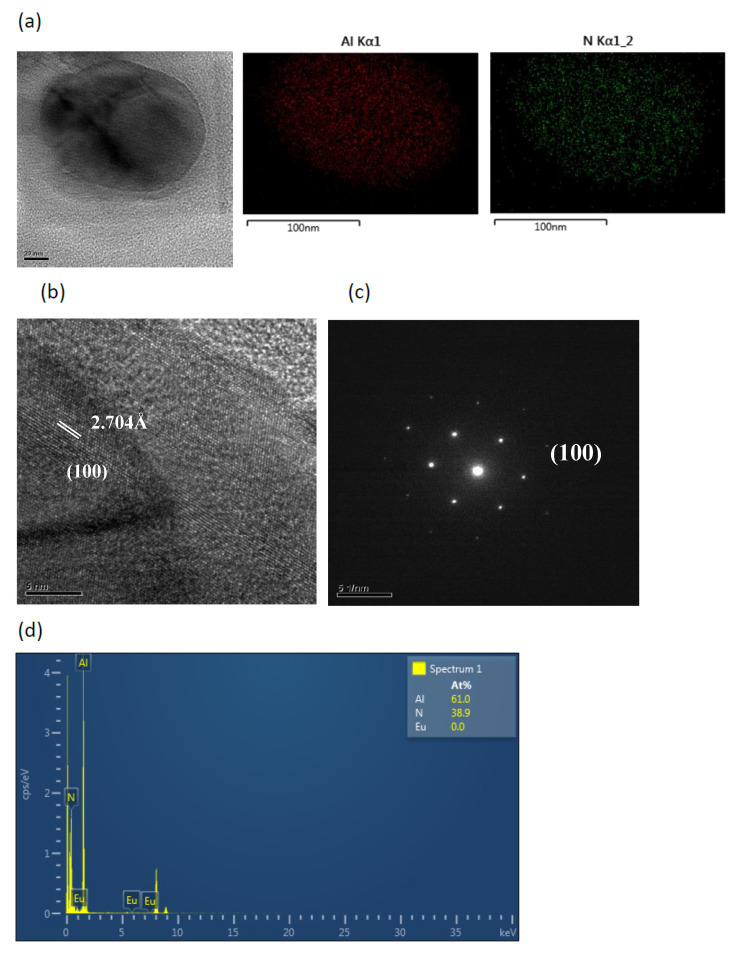
(**a**) TEM image and corresponding element mapping of Al and N; (**b**) HRTEM image; (**c**) SAED pattern; and (**d**) element analysis of a plate-like crystal (showing it to be AlN) in a product synthesized with Ca:Al:Si:Si_3_N_4_:Eu_2_O_3_:NaN_3_:NH_4_Cl = 1.0:1.0:0.25:0.25:0.02:3.5:0.6.

Figure 3a shows the TEM image and element mapping of Ca, Al, Si, N, and Eu for the bar-like CaAlSiN_3_:Eu^2+^ in the products synthesized with Al = 1.0. The mapping shapes of the five elements are consistent with that of the TEM image. The d-spacings directly measured from the HRTEM (high resolution transmission electron microscopy) image in Figure 3b is 4.839 Å, which can be indexed to the (110) plane. The corresponding SAED pattern and TEM-EDS are shown in Figure 3c,d, respectively. Figure 4a shows a TEM image for the agglomerated fine CaAlSiN_3_:Eu^2+^ particles in the products synthesized with Al = 1.0. The d-spacing in HRTEM image in Figure 4b is 3.482 Å which can be indexed to the (111) plane. Figure 5a shows the TEM image and element mapping of Al and N for the plate-like AlN crystal in the products synthesized with Al = 1.0. The mapping shapes of the two elements are consistent with that of TEM image. The d-spacings directly measured from the HRTEM images in Figure 5b are 2.704 Å, which can be indexed to the (100) plane. The corresponding SAED pattern and TEM-EDS are shown in Figure 5c,d, respectively. 

### 2.2. Effects of Al Content on Photoluminescence Properties

**Figure 6:**
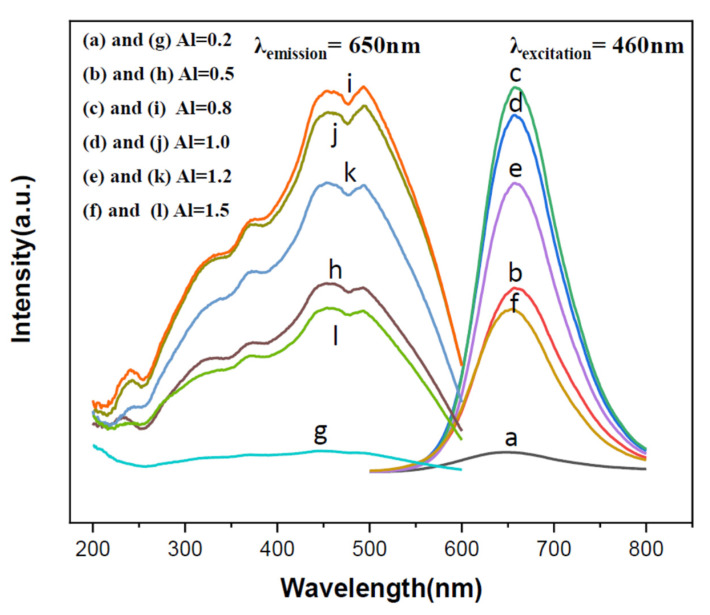
The emission (**a**–**f**) and excitation (**g**–**l**) spectra of the phosphors synthesized in this study.

Figure 6 shows the excitation and emission spectra of the products synthesized with various Al contents. The excitation spectra were obtained by measuring the emission at 650 nm and the emission spectra were measured by excitation at 460 nm. As can be seen, the wavelength regions of the excitation spectra are all similar (from ~225 to ~600 nm) for various Al contents and their emission intensity increases as the Al content is increased from 0.2 to 0.8, but decreases as the Al content is further increased from 0.8 to 1.50. 

**Figure 7:**
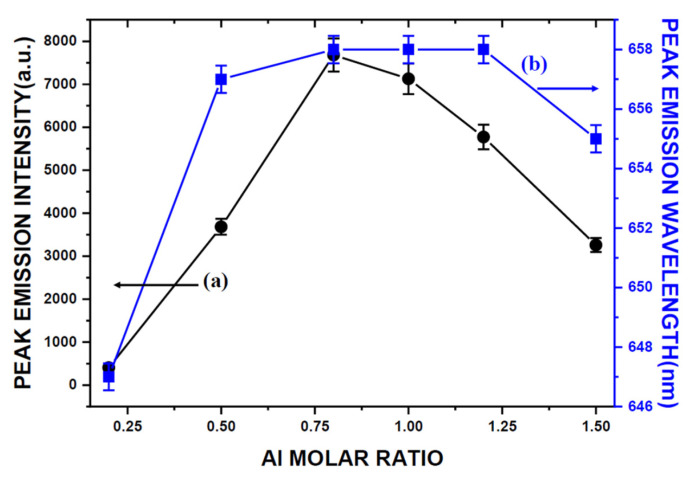
Dependence of (**a**) peak emission intensity and (**b**) peak emission wavelength on the Al molar ratio.

The dependence of the peak emission intensity and wavelength on the Al molar ratio is shown in Figure 7. The peak emission intensity increases with increasing Al molar ratio to a maximum at the molar ratio of 0.8 and begins to decrease, with further increase at the molar ratio of 1.5. The peak emission wavelength increases from 647 to 658 nm as the Al molar ratio is increased from 0.2 to 1.2 and begins to decrease, with further increase at the Al molar ratio to 1.5.

As mentioned previously, the XRD measurements (Figure 1) show the phases of CaSIN, AlN, and residual Si that were detected. The XRD peak intensity of CASIN increased with increasing Al to a maximum at Al = 1.2, indicating that the formation of CASIN continuously increases as the Al content is increased from 0.2 to 1.2 and thus the peak emission wavelength (as shown in Figure 7) increased from Al = 0.2 to Al = 1.2. However, the peak emission wavelength decreases as the Al molar ratio is further increased from Al = 1.2 to Al = 1.5, which resulted from the large amount of AlN produced in the as-synthesized product. K. Inoue et al., N. Hirosaki, B. Dierre, and E. Kuokstis [7,8,9,10] reported the AlN was to give UV emission. Thus, the peak emission wavelength of the as-synthesized product will decrease with the increase of AlN in the product. S-X. Li et al. [4] also reported that the AlN-like secondary phase was always formed according to the stoichiometric composition with a Ca/Al/Si molar ratio of 1:1:1 to prepare CaAlSiN_3_-based red-emitting phosphors, even at high temperature and pressure, because of the low solubility of Al in CaAlSiN_3_. As can be seen in Figure 7, the emission intensity increases with increasing Al from Al = 0.2 to Al = 0.8, and with Al further increasing from Al = 0.8 to Al = 1.5, the emission intensity decreases. One of the reasons is that large amounts of AlN produced in the as-synthesized products, such as Al > 0.8, cause the emission intensity to decrease. Another of the reasons is the morphology of the as-synthesized products transformed. Chung et al. [2] reported that the three major types of morphology were observed for the synthesized products: plate-like crystals, bar-like crystals, and agglomerated fine particles. Among the three types of morphology of CASIN, the bar-like morphology seems to give a stronger emission than the other two. As can be seen in Figure 2, the morphology of the as-synthesized products changes gradually from plate-like crystals to bar-like crystals as Y increase from Al = 0.2 to Al = 0.8 and as Al is further increased from Al = 0.8 to Al = 1.5, the morphology of the as-synthesized products changes gradually from bar-like crystals to agglomerated fine particles and plate-like crystals. Therefore, we concluded that the bar-like crystals of CaAlSiN_3_:Eu^2+^ have stronger emission than the other two morphologies (i.e., plate-like and agglomerated fine particles of CaAlSiN_3_:Eu^2+^), thus leading to the above-mentioned variation of the emission intensity from Al = 0.8 to Al = 1.5. In the next article, we will use a synchrotron source and X-ray nanoprobe of national synchrotron radiation research center (NSRRC) to provide the obvious and direct proof to prove the relationship of particle’s morphology and emission intensity, as we describe in this article.

**Figure 8:**
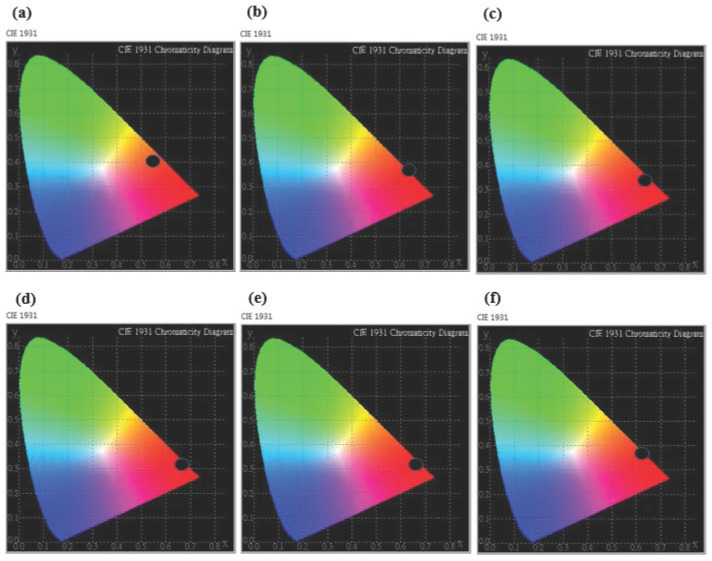
The CIE chromaticity coordinates of CaAlSiN_3_:Eu^2+^ phosphors doped with 0.02 molar ratio of Eu^2+^ under the excitation of 460 nm with Al = 0.2 (**a**); 0.5 (**b**); 0.8 (**c**); 1.0 (**d**); 1.2 (**e**); and 1.5 (**f**).

Figure 8 shows the CIE chromaticity coordinates of CaAlSiN_3_:Eu^2+^ phosphor synthesized with various Al contents and doped with 0.02 molar ratio of Eu under the excitation of 460 nm. It can be seen that the CaAlSiN_3_:Eu^2+^ phosphor can be tuned from orange to the red color, which corresponds to the chromaticity coordinates (x, y) varying from point A (0.5630, 0.4015) at the Al molar ratio of 0.2 to point B (0.6210, 0.3634) at the Al molar ratio of 1.5. These results indicate that CaAlSiN_3_:Eu^2+^ phosphor which, when synthesized by the combustion synthesis method, is a promising orange-red to red phosphor that can be applied for industrial application with mass production for white LED lighting.

## 3. Materials and Methods

The present study was carried out by employing the combustion synthesis process for the synthesis of CASIN. Calcium (Alfa Aesar, Heysham, UK), aluminum (First Chemical Work, Taipei, Taiwan), silicon (Alfa Aesar, Ward Hill, MA, USA), europium oxide (Baogang Group, Baotou, China), sodium azide (Johnson Matthey, Tokyo, Japan), ammonium chloride (Panreac, Barcelona, Spain), and silicon nitride powders (Seminc Company, Ward Hill, MA, USA) were used as the starting materials. To study the effect of Al content on the formation of CASIN and its photoluminescence property, the molar ratio of Al varies from 0.2 to 1.5, while those of others were kept constant as Ca:Si:Si_3_N_4_:Eu_2_O_3_:NaN_3_:NH_4_Cl = 1:0.25:0.25:0.02:3.5:0.6. These starting materials were thoroughly mixed in the desired proportions and then pressed into cylindrical compacts (referred to as reactant compacts) with 17 mm in diameter and ~16 mm in length. The reactant compact thus obtained was then wrapped up with an igniting agent (i.e., a mixed powder of Mg and Fe_3_O_4_ at 4:1 molar ratio) to obtain a larger cylindrical compact (referred to as a wrapped compact) with 30 mm in diameter and ~30 mm in length.

The combustion synthesis reactor used in this study has been described and shown schematically in our previous studies [11] and thus is not repeated here. The wrapped compact was placed on a height adjustable stage, which was adapted so that the top surface of the compact was about 5 mm below the tungsten heating coil. The reactor was evacuated to 65 Pa by flushing with nitrogen between the evacuations. After the evacuation, the reactor was backfilled with nitrogen to the desired pressures. The combustion reaction was ignited by heating the top surface of the compact for ~10 s by applying an electrical power of ~1 KW to the heating coil [1,2]. After combustion, the igniting agent was converted to MgO + Fe, which was loosely attached to the interior product. The interior product could thus be easily separated from the combustion product of the igniting agent. The as-synthesized products contained byproducts AlN and unreacted Si. 

The crystalline phase of the product was identified by X-ray diffraction (D8 Discover, Bruker Axs Gmbh, Karlsruhe, Germany) using Cu Kα radiation operating at 40 kV and 40 mA. The powder diffraction patterns were obtained using a Bruker Axs Nanostar Universal System coupled with an IuS-type X-ray tube for a microfocus X-ray source with a wavelength of 0.154 nm. The diffraction signals were recorded on an image plate with an exposure period of 30 min. The data was collected in the range of 20° ≤ 2θ ≤ 80°. Scanning electron micrographs (SEM), together with energy-dispersed X-ray spectroscopy (EDS) measurements, were performed on a field-emission scanning electron microscope (SU-8100, Hitachi, Japan). The excitation and emission spectra were measured at room temperature using a Fluorescent Spectrophotometer (F-7000, Hitachi, Japan) with a 150 W xenon lamp at a scanning speed of 1200 nm/min. High resolution transmission electron microscopy (i.e., HRTEM) images, selected area electron diffraction (SAED) patterns, and element mapping were obtained on a transmission electron microscope (JEM-2100F, JEOL, Tokyo, Japan) equipped with an energy-dispersive X-ray spectroscope (EDS) system operating at 200 kV.

## 4. Conclusions

Al content in the reactant mixture was found to significantly affect the formation and photoluminescence properties of CaAlSiN_3_:Eu^2+^ phosphor. The formation of CASIN continuously increases as the Al content is increased from 0.2 to 1.2, and thus the peak emission wavelength increases from 647 to 658 nm. However, when the Al molar ratio is further increased to Al = 1.5, the peak emission wavelength decreases, which results from the large amount of AlN produced in the as-synthesized product. In addition, the peak emission intensity increases with increasing Al molar ratio to a maximum at Al = 0.8 and begins to decrease with further increase to Al = 1.5. One of the reasons is that large amounts of AlN produced in the as-synthesized products as Al > 0.8 and thus the emission intensity decreases. Another of the reasons is that the morphology of the as-synthesized products is transformed. Three major types of morphology were observed for the synthesized products: bar-like crystals, plate-like crystals, and agglomerated fine particles. Among the three types of morphology of CASIN, the bar-like crystals give a stronger emission than the other two. In the next article, we will use a synchrotron source and X-ray nanoprobe of national synchrotron radiation research center (NSRRC) to provide the obvious and direct proof to prove the relationship of particle’s morphology and emission intensity, as we describe in this article. The CIE chromaticity coordinates and the exterior color of the synthesized CASIN phosphors are similar with the commercial product, and thus can be applied for industrial application with mass production for white LED lighting.
